# *Moringa oleifera* Leaves’ Extract Enhances Nonspecific Immune Responses, Resistance against *Vibrio alginolyticus*, and Growth in Whiteleg Shrimp (*Penaeus vannamei*)

**DOI:** 10.3390/ani12010042

**Published:** 2021-12-26

**Authors:** Zaenal Abidin, Huai-Ting Huang, Zhen-Hao Liao, Bo-Ying Chen, Yu-Sheng Wu, Yu-Ju Lin, Fan-Hua Nan

**Affiliations:** 1 Department of Aquaculture, National Taiwan Ocean University, No. 2, Pei-Ning Road, Keelung 20224, Taiwan; zaenalabidin@unram.ac.id (Z.A.); 29934001@mail.ntou.edu.tw (H.-T.H.); smallhowhow@mail.ntou.edu.tw (Z.-H.L.); 20934001@mail.ntou.edu.tw (B.-Y.C.); 2 Department of Aquaculture, National Pingtung University of Science and Technology, No. 1, Xue-Fu Road, Pingtung 912301, Taiwan; wuys0313@mail.npust.edu.tw; 3 Department of Life Sciences, National Chung Hsing University, No. 145, Xing-Da Road, South District, Taichung City 40227, Taiwan; yjl@dragon.nchu.edu.tw

**Keywords:** challenge trial, gene expression, herb, innate immunity, weight gain

## Abstract

**Simple Summary:**

This study found that moringa (*Moringa oleifera*) leaves’ water extract triggered phenoloxidase activity, phagocytic rate, and superoxide anion production in whiteleg shrimp (*Penaeus vannamei)* hemocytes by an in vitro assay. By an in vivo assay, a dietary moringa extract enhanced the total hemocyte count, phenoloxidase activity, phagocytic rate, immune-related gene expressions, and growth performance of the whiteleg shrimp. The administration of dietary moringa extract increased the survival rate after challenging the whiteleg shrimp with *Vibrio alginolyticus*.

**Abstract:**

Moringa is widely known as a plant with high medicinal properties. Therefore, moringa has a high potential for use as an immunostimulant in shrimp. This study investigated the effect of a moringa water extract on the immune response, resistance against *V. alginolyticus*, and growth performance of whiteleg shrimp. To perform the in vitro assay, hemocytes were incubated with different concentrations of the moringa extract. Furthermore, the moringa extract was incorporated at 0 (control), 1.25 g (ME1.25), 2.5 g (ME2.5), and 5.0 g (ME5.0) per kg of diet for the in vivo assay. During the rearing period, immune responses, namely the total hemocyte count (THC), phenoloxidase (PO) activity, phagocytosis activity, superoxide anion production, and immune-related gene expression were examined on days 0, 1, 2, 4, 7, 14, 21, and 28. Growth performance was measured 60 days after the feeding period. Furthermore, the shrimp were challenged with *V. alginolyticus* after being fed for different feeding durations. The results of the in vitro assay revealed that 100–250 ppm of the moringa extract enhanced the PO activity, phagocytic rate (PR), and superoxide anion production. The findings of the in vivo assay demonstrated that the THC, PO activity, PR, and immune-related gene expression, including alpha-2-macroglobulin, prophenoloxidase II, penaeidin2, penaeidin3, anti-lipopolysaccharide factor, crustin, lysozyme, superoxide dismutase, and clotting protein, were higher in the group of ME.25 and ME5.0 than in the control and ME1.25 at several time points. Growth performance was significantly increased (*p* < 0.05) in the ME2.5 group compared to the control group. Furthermore, the dietary ME2.5 resulted in a higher survival rate compared to that of the control group after challenging with *V. alginolyticus*, especially at ME2.5 administered for 4 and 7 days. This study indicated that the incorporation of the moringa extract at 2.5 g per kg of diet enhanced the immune response, the growth performance of the whiteleg shrimp, and the resistance against *V. alginolyticus* infection.

## 1. Introduction

The intensification in shrimp farming has been associated with disease outbreaks [1]. Shrimp live under various stress conditions, related to the high stocking density and poor water quality, making them highly susceptible to diseases such as vibriosis [2,3]. Their susceptibility to *Vibrio alginolyticus* may increase when the water quality is not suitable for [4,5,6], resulting in high mortality and, finally, considerable economic losses in aquaculture. To maintain the shrimp’s health during the grow-out period, aquaculturists have developed a cost-effective healthy diet by using immunostimulants. Herbal immunostimulants are a promising alternative, not only because their antimicrobial properties are cost-effective and eco-friendly, with negligible side effects, but also because they improve the growth performance [7,8].

Moringa (*Moringa oleifera*) is widely known for its nutritional value. Moringa is a good source of fibers, proteins, vitamins, minerals, and lipids [9]. Moringa possesses many pharmacological properties, such as anticancer, antidiabetic, anti-inflammatory, antioxidant, antifungal, and antibacterial properties associated with the presence of bioactive compounds, including phenolic acid and flavonoids [10,11,12].

Aquatic animal studies have indicated that incorporating a moringa leaves’ extract in the animals’ diet could improve their growth and physiology and upregulate immune-related gene functions. A 0.5% moringa extract in the diet increased the level expression of immune-related genes, the resistance against *V. anguillarum*, and the growth of giant freshwater prawns (*Macrobranchium rosenbergii*) [13], whereas a 1% extract increased the antioxidant activities and resistance against the *Photobacterium damselae* infection of whiteleg shrimp (*Penaeus vannamei*) [14]. Moringa leave powder supplementation at 1.5 to 5% in diet effectively enhanced the immunity and controlled infection in nile tilapia (*Oreochromis niloticus*) [15], and at 20% in diet it enhanced the antioxidant activity and immune parameters of rainbow trout (Oncorhyncus mykiss) [16]; moreover, at 15% in diet it increased the growth and immune responses of grass carp (Ctenopharyngodon idella) [17]. In aqueous extract form, at 600 ppm, it induced antioxidant and antigenotoxic effects in nile tilapia [18], and at 1000 ppm it increased the resistance of whiteleg shrimp against *V. harveyi* [19]; moreover, mixing at 20 mL per kg diet improved the immunity and stress resistance of common carp (*Cyprinus carpio*) [20]. Despite its numerous advantages, moringa also has toxicological properties, which showed a dose-response relationship in some species [16,21,22,23]. The concentration of moringa that is safe for whiteleg shrimp hemocytes can become known through a viability assay. The immune system of the shrimp requires hemocytes (for encapsulation, nodule formation, and phagocytosis), several plasma components (antimicrobial peptide, histone, lysosomal enzyme, lipopolysaccharide, glucan binding protein, and recognition molecules), and a multimeric system (clotting protein cascade, prophenoloxidase system) [24], in which the functions of the immune cells are regulated through the expression of immune genes. Moringa’s stimulation of the immune mechanisms is expected to increase whiteleg shrimp’s ability against *V. alginolyticus*. However, the application of moringa should not retard the growth, because some herbs might suppress growth while increasing immunity, or vice versa [25,26,27].

Some studies have investigated the effect of moringa extracts on fish and shrimp; however, inadequate research is available on the effect of moringa extracts in diet as in vitro and in vivo immunostimulants for whiteleg shrimp. The aim of this study was therefore to investigate the effect of the moringa leaves’ water extract on the viability and activation of the phenoloxidase, phagocytosis, and superoxide anion generation on hemocytes in vitro and in vivo. In addition, this study analyzed the effect of the extract on the expression of immune-related genes, the resistance against *V. alginolyticus*, and the growth performance of whiteleg shrimp.

## 2. Materials and Methods

### 2.1. Moringa Preparation

*Dry* moringa leaves were a commercial product from Rumah Kelor (Blora, Indonesia). Dried leaves were milled into a fine powder and mixed with hot distilled water (98 °C) at a ratio of 1:9 and subsequently left for 24 h at room temperature. The resulting solution was filtered using a muslin cloth and Whatman filter paper no. 1 to separate the solids and the liquid. For the lyophilization, the liquid was frozen at −80 °C and dried in a freeze dryer for three days to yield extract powder [20]. The recovery rate of dry leaves was 13.1%. The powder extract was stored at −20 °C until further use.

### 2.2. Whiteleg Shrimp

The shrimp was obtained from the Aquatic Animal Center, National Taiwan Ocean University (NTOU), Taiwan. They were acclimated to laboratory conditions at a temperature of 28 °C ± 1 °C and a salinity of 32 ± 1 ppt in a circular tank for two weeks. The shrimp were fed a commercial diet three times a day during the acclimation period. Only healthy-appearing intermolt-stage shrimp (no disease signs, normal feeding behavior, hard carapace) were selected for use in subsequent treatments.

### 2.3. In Vitro Study of Viability and Immune Response

A moringa extract and hemolymph were prepared following previous studies [28,29]. The moringa extract was diluted in different solutions to get concentrations of 0, 25, 100, 250, 500, 1000, 1500, and 2000 mg per L. The solution for the viability, phagocytosis, and superoxide anion$\;\left(O_{2}^{-}\right)$ production assay was modified with a complete Hank’s balanced salt solution (MCHBSS; 10 mM calcium chloride, 3 mM magnesium chloride, 5 mM magnesium sulfate in HBSS (Gibco, Bleiswijk, Netherlands)), and for phenoloxidase the activity assay was a cacodylate buffer (sodium cacodylate 0.01 M (Sigma-Aldrich^®^, St. Louis, MO, USA), sodium chloride 0.45 M (Fluka^TM^, Seelze, Germany)), and trisodium citrate 0.1 M (Merck, Darmstadt, Germany).

Three shrimp (15 ± 0.2 g) were used for the in vitro study. Hemolymph was withdrawn from the ventral sinus, and then mixed with an anticoagulant (30 mM trisodium citrate, 0.34 M sodium chloride, 10 mM EDTA (Riedel-de Haen^TM^, Seelze, Germany), 0.12 M glucose (Fluka^TM^), pH 7.4) at a ratio of 1:9. The hemolymph-anticoagulant mixture was dropped on a hemocytometer (Marienfeld^TM^, Marienfeld, Germany) to count the number of hemocytes. The hemocytes were adjusted to 1 × 10^6^ cells per mL. Subsequently, 100 µL of diluted hemolymph was distributed into 96 wells (Simply^TM^, Taoyuan, Taiwan). The hemolymph was centrifuged (at 800× *g* for 20 min, at 4 °C), and then the supernatant was removed. A hemocyte pellet was then added with different concentrations of moringa extract and incubated for 30 min. These treated hemocytes were used for the viability, phenoloxidase, and superoxide anion production.

Viability was measured following a previously reported method [30] with some modifications. A 100-µL hemocyte treated with different concentrations of moringa extract was centrifuged at 800× *g* for 20 min, at 4 °C, and then the supernatant was removed. A total of 100 µL of 3-[4, 5-dimethylthiazol-2-yl]-2, 5 diphenyl tetrazolium bromide (MTT; Sigma-Aldrich^®^, St. Louis, MO, USA) solution (0.5 mg of MTT/mL of MCHBSS]) was added to the hemocytes and incubated for 4 h in the dark. After incubation, the MTT formazan product was dissolved in 100 µL of dimethyl sulfoxide (DMSO) (Riedel-de Haen^TM^, Seelze, Germany). The absorbance was immediately recorded at 570 ƞm using a spectrophotometer (Model U-2000 Hitachi, Tokyo, Japan), and the percentage of viability was calculated using the following formula:

Cell viability (%) = ([Absorbance of the control − Absorbance of the sample]/Absorbance of the control) × 100.

Phenoloxidase (PO) activity was spectrophotometrically measured by recording the formation of dopachrome, which resulted from L-dihydroxyphenylalanine (L-DOPA) by using a previously reported method [31] with some modifications. A volume of 100 µL of hemocytes treated with different concentrations of moringa was centrifuged at 800 × *g* for 20 min, at 4 °C. The supernatant was removed, and the hemocyte was resuspended with 100 µL of cacodylate buffer. The hemocyte was then subjected to a freeze–thaw cycle to induce cell lysis. Hemocyte lysate was centrifuged at 800 × *g*, at 4 °C, for 20 min. Subsequently, the hemocyte lysate supernatant was placed in a 96-well plate and treated with 50 µL of trypsin (Sigma-Aldrich^®^, St. Louis, MO, USA) and 50 µL of L-DOPA (Sigma-Aldrich^®^, St. Louis, MO, USA). The absorbance was measured at 492 nm.

Phagocytosis activity was examined following a previous method [32] with some modifications. Briefly, a 100-µL hemolymph-anticoagulant mixture (1 × 10^6^ cells per mL) was dropped on a glass slide and allowed to adhere for 1 h. A volume of 100 µL of the moringa extract with different concentrations was added to the hemocytes’ monolayer and incubated for 1 h. Then, 100 µL of latex beads (0.8 µm, 3 × 10^7^ beads per mL, Sigma-Aldrich^®^, St. Louis, MO, USA) was added to the hemocytes and set for 60 min. Subsequently, the hemocytes were washed with MCHBSS. The cells were fixed with 100% methanol and then stained with a 5% Giemsa solution (Sigma-Aldrich^®^, St. Louis, MO, USA). A total of 100 cells were observed under a light microscope (Olympus, Tokyo, Japan). The phagocytic rate (PR) and phagocytic index (PI) were calculated using the following formula:

PR (%) = (phagocytic hemocytes/total hemocytes) × 100; PI = beads per phagocytic cell/number of phagocytic cells.

Superoxide ($O_{2}^{-}$) production was evaluated by performing the nitroblue tetrazolium (NBT) reduction assay as described previously [33] with some modifications. Briefly, 100 µL of hemocytes treated with different concentrations of moringa extract was centrifuged at 800× *g* for 20 min, at 4 °C. After removing the supernatant, 100 µL of zymosan (Sigma-Aldrich^®^, St. Louis, MO, USA) was added to induce superoxide anion production, followed by staining with NBT (Panreac, Darmstad, Germany) solution for 30 min. Subsequently, 100% methanol was added to terminate the staining reaction, and the hemocytes were cleaned with 70% methanol three times. To dissolve the formazan present in the cytoplasm, 120 µL of 2 M KOH (Sigma-Aldrich^®^, St. Louis, MO, USA) and 140 µL of DMSO were added. The absorbance was read at 630 nm.

### 2.4. Diet Preparation

Four types of diet (three treatment diets and one control diet) were formulated as described by Ngo et al. [28]. The experimental diet was prepared by adding the moringa extract along with the ingredients listed in Table 1 at concentrations of 1.25, 2.50, and 5.0 g per kg of feed (hereafter referred to as ME1.25, ME2.5, and ME5.0, respectively). The control diet was prepared using the same ingredients but without the moringa extract. The prepared diets were dried at 35 °C for 24 h and then stored at 4 °C for further use. The contents of crude protein, lipid, moisture, and ash in the diets were analyzed using standard methods [34].

### 2.5. In Vivo Study of the Immune Response

A total of 96 shrimp (15 ± 1 g) were randomly distributed into 12 60-L tanks with eight shrimp per tank, corresponding to four experimental groups with three replications and eight sampling days. The shrimp were maintained in a recirculation culture system with a 50% water exchange per day. They were fed the experimental diets (ME1.25, ME2.5, and ME5.0) or the control diet daily at 09.00, 15:00, and 21:00 at a feeding rate of 5–8% of body weight. Three shrimp were collected from each group at 0, 1, 2, 4, 7, 14, 21, and 28 days in order to analyze all the immune parameters and related gene expressions. The hemolymph was withdrawn from the ventral sinus using 1 mL sterile syringes with anticoagulant inside and divided into two tubes—one tube for the immune assay and another tube for the immune-related gene expression assay.

The density of hemocytes for the in vivo response immune assay was counted for the total hemocyte count (THC) and then adjusted to 1 × 10^6^ cells per mL. A total of 100 µL of the mixture hemolymph-anticoagulant was centrifuged at 800× *g* for 20 min, at 4 °C, and then the supernatant was removed. The PO activity, phagocytosis activity, and superoxide anion production were measured as described in Section 2.3, without prior exposure to different concentrations of moringa extract.

### 2.6. Immune-Related Gene Expression

The hemocytes of the shrimp were individually lysed using the TriZol^TM^ reagent (Invitrogen, Carlsbad, CA, USA) and then subjected to chloroform (Macron, PA, USA) extraction according to the manufacturer’s instructions. The mixture was centrifuged at 12,000 × *g* for 15 min at 4 °C. The colorless upper aqueous phase was transferred into a new tube. After the addition of isopropanol (Sigma-Aldrich^®^, St. Louis, MO, USA) to this tube, the mixture was centrifuged at 12,000 × *g* for 15 min at 4 °C. The pellet was washed with 75% ethanol and then air dried. Subsequently, the pellet was dissolved in diethylpyrocarbonate water (Thermo Fisher Scientific, MA, USA) and quantified with a spectrophotometer (QuickDrop^TM^ Micro-Volume spectrophotometer, CA, USA).

The extracted RNA was then treated with DNase (Bionovas^®^, Toronto, ON, Canada) at 37 °C for 30 min and 70 °C for 5 min, to inactivate the DNase. The DNase-treated total RNA was denatured by heating at 65 °C for 5 min in 12 µL of ddH_2_O containing oligo dT_(18)_ as the primer and 2× Fast premix (Bionovas^®^, Toronto, ON, Canada). First-strand cDNA was synthesized by adding 1 µL of HiScript 1 reverse transcriptase (Bionovas^®^, Toronto, ON, Canada). The reaction was performed at 42 °C for 30 min and then terminated by heating at 85 °C for 5 min.

Nine immune-related genes including prophenoloxidase2 (*ProPO2*), α 2-macroglobulin (*A2M*), penaeidin2 (*Pen2*), penaeidin3 (*Pen3*), anti-lipopolysaccharide factor (*ALF*), crustin (*Crus*), lysozyme (*Lyz*), superoxide dismutase (*SOD*), and clotting protein (*CP*) were analyzed using the primers listed in Table 2. A quantitative polymerase chain reaction (PCR) was performed using a real-time PCR system (Applied Biosystems^®^, Waltham, MA, USA) with SYBR Green. The amplification was performed in a 96-well plate in a 20-µL reaction mixture containing 0.4 µL of the 10-nm reverse and forward primer, 2 µL of 10-ng cDNA template, 10 µL of SYBR Green 2× Master Mix (Agilent Technologies, Santa Clara, CA, USA), and 7.2 µL of ddH_2_O. The PCR was performed at 95 °C for 30 s, followed by 40 cycles at 95 °C for 15 s, and 60 °C for 1 min. The expression level of the gene of interest was normalized to the expression level of elongation factor-1α (*EF-1α*). The relative expression level of interested genes was calculated following the previous method by Livak and Schmittgen [35].

### 2.7. Growth Performance

A total of 120 healthy shrimp with an average weight of 0.59 ± 0.05 g were randomly assigned to four groups with three replicates. Each replication included ten shrimp maintained in a 60-L tank with a recirculation system, and the water exchange was 50% per day. The shrimp were fed 5–8% of their body weight three times a day for 60 days. Each shrimp was weighed every two weeks, and the amount of diet was adjusted accordingly. The water quality was monitored regularly. The dissolved oxygen concentration was maintained above 6 mg per L. The temperature, salinity, and pH varied from 28 to 29 °C, 30 to 33‰, and 6.8 to 7.5, respectively. At the end of the feeding trial, the following growth performance parameters were calculated using the formula given below.

Weight gain (%) = ([final weight (g) − initial weight (g)]/initial weight (g)) × 100

Feed conversion rate (FCR) = (consumed diet (g)/biomass gain (g)) × 100

Specific growth rate (SGR; % per day) = ([ln final weight (g) − ln initial weight (g)]/time (days)) × 100

Survival rate (%) = ([number of individuals at the end of the evaluation period/initial number of individuals stocked]) × 100

### 2.8. Challenge with V. alginolyticus

A total of 600 healthy shrimp with an average weight of 16 ± 1.0 g were divided into four groups (unchallenged control, challenged control, ME2.5, and ME5.0) of 150 shrimp each. Each group was further divided into five subgroups based on the different day duration of diet administrations (on days 1, 2, 4, 7, 14) before being injected with *V. alginolyticus*. The shrimp were continuously fed with an experimental diet for three days after vibrio injection. Each subgroup had three replications, with ten shrimp for each replication.

For the bacteria preparation, *V. alginolyticus* was provided by Professor Liu Ping-Chung’s laboratory (Department of Aquaculture, NTOU Taiwan). The stocks were spread on a thiosulphate–citrate–bile salts–sucrose agar (TCBS, Neogen) supplemented with 2% NaCl for 8 h at 26 °C. One colony was transferred to a 10-mL tryptic soy broth (TSB, Neogen) supplemented with 2% NaCl. The TSB was incubated for 4 h at 26 °C and then centrifuged at 7155× *g* for 20 min at 4 °C. The supernatant was removed, and the bacterial pellet was resuspended in a saline solution (0.85% NaCl) to obtain an optical density of 0.590 at 600 nm, equivalent to 1.09 × 10^7^ colony-forming units (CFUs) per mL. The shrimp were injected with 20 µL of the bacterial suspension into the ventral sinus. The dosage of the bacterial suspension received was 2 × 10^5^ CFU per shrimp. The survival rate of the shrimp was calculated at 6, 12, 24, 48, and 72 h after the injection.

### 2.9. Statistical Analysis

All data are presented as mean ± standard deviation. The experiments were designed according to a completely randomized design. Immune parameters (in vitro and in vivo), immune-related gene expression, growth performance, and survival rate were tested with Shapiro–Wilk and Levene’s tests for normality and homogeneity of variance. Percentage data of survival rate were transformed with the arcsin function. The differences between treatments were determined using a one-way ANOVA followed by a Tukey’s test. Statistical significance was assumed for a *p*-value lower than 0.05. Statistical analyses were performed using the IBM SPSS Statistic version 25 software.

## 3. Results

### 3.1. In Vitro Study of Viability and Immune Response

**Figure 1:**
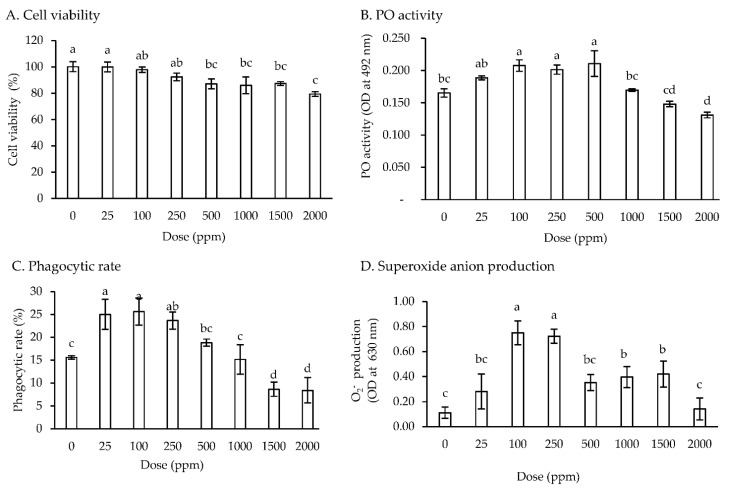
In vitro assay. The effect of different concentrations (0, 25, 100, 250, 500, 1000, 1500, and 2000 ppm) of moringa extract on cell viability, PO (phenoloxidase) activity, phagocytic rate, and superoxide anion production in the hemocytes of whiteleg shrimp. The values represent the means. The error bars indicate ± standard deviation (*n* = 3). Bars with different letters are significantly different (*p* < 0.05).

No significant difference with controls in the cell viability of hemocytes was observed when treated with 25–250 ppm of the moringa extract. When hemocytes were treated with 500 to 2000 ppm of the moringa extract, their viability decreased; however, the viability was >80% for all treatment concentrations (Figure 1A). A higher PO activity was observed in hemocytes treated with 100–500 ppm of the moringa extract than in controls and in those treated with 1000–2000 ppm of the moringa extract (Figure 1B). Compared with the control hemocytes, PR was significantly increased (*p* < 0.05) to 23.7–25.0% in hemocytes treated with 25–250 ppm. Subsequently, the PR was gradually decreased to 8.4% with the increasing concentration of the moringa extract (Figure 1C). No significant difference in the PI was observed in hemocytes treated with 0–500 ppm of the moringa extract (data not shown). The superoxide anion production was higher in hemocytes treated with 100 and 250 ppm moringa extract than in those treated with 0, 25, 500, 1000, 1500, and 2000 ppm of the moringa extract (Figure 1D).

### 3.2. In Vivo Study of Immune Responses

**Figure 2:**
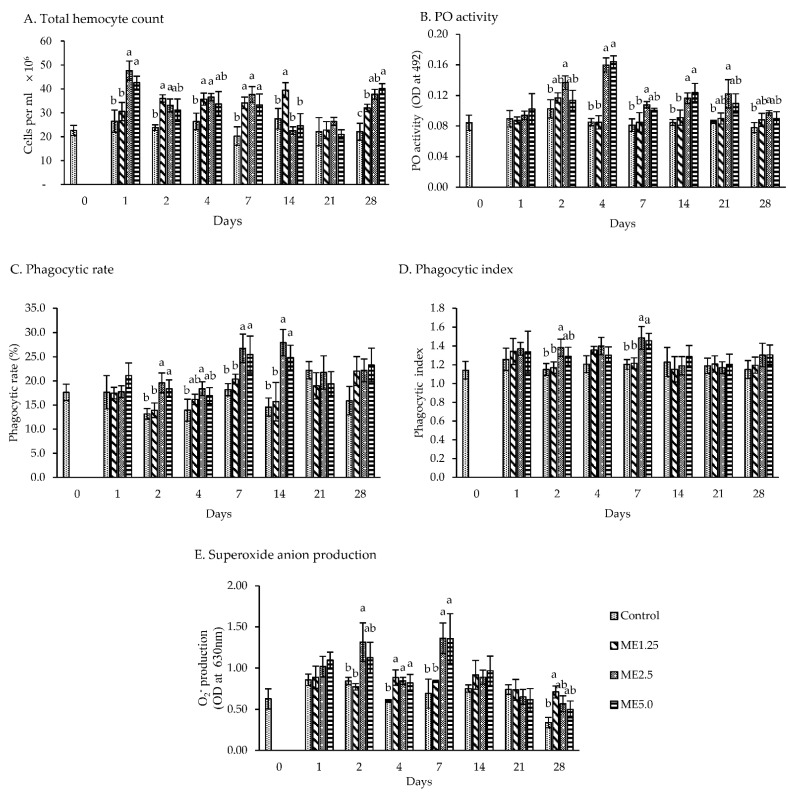
In vivo immune assay. The effect of different concentrations of moringa extract in diet (control, ME1.25, ME2.5, and ME5.0) on the total hemocyte count, phenoloxidase (PO) activity, phagocytic rate, phagocytic index, and superoxide anion production in the hemocytes of whiteleg shrimp. The values represent means. The error bars indicate ± standard deviation (*n* = 3). Bars with different letters at the same time point are significantly different (*p* < 0.05).

The THC was consistently lower (*p* < 0.05) in the control group than in ME2.5 on days 1–7 and 28. The THC in ME2.5 on days 1–7 was 34 × 10^6^ cell per mL, on average, while it was 24 × 10^6^ cells per mL, on average, in the control group. The THC did not significantly differ (between the control group and the dietary moringa groups on day 21 (Figure 2A)).

The PO activity (Figure 2B) on day 1 did not significantly differ between treatment groups. The PO activity was higher in the ME2.5 group from day 2 to day 28 than in the control group, while ME5.0 was higher than the control group on days 4 and 14. On days 4, the PO showed a two-times increase in ME2.5 and ME5.0. Furthermore, the PO activity did not significantly differ between the ME2.5 and ME5.0 groups in all days.

The PR was higher in the ME2.5 group than in the control group on days 2, 4, 7, and 14 (*p* < 0.05). However, no significant difference was found between ME2.5 and ME5.0 in all days. The highest PR was noted on days 7 and 14 in the ME5.0 and ME2.5 groups, ranging from 24.8 to 27.9%, while the control group ranged only from 14.6 to 18.2% (Figure 2C). The PI was not higher than the control group in all days of observation, except in the ME2.5 group (*p* *<* 0.05) on days 2 and 7. However, on these days, ME2.5 and ME5.0 were not significantly different (Figure 2D).

Superoxide anion production was higher (*p* < 0.05) in the ME2.5 group than in the control group on days 2, 4, and 7, and not significantly different (*p* < 0.05) compared to ME5.0. The superoxide anion production doubled on day 7 in the ME2.5 and ME5.0 groups compared to the control group. No significant difference was found in the superoxide anion production on days 1,14, and 21 between the treatment and control groups, while the ME1.25 group exhibited a higher superoxide anion production compared with the control group on days 4 and 28 (Figure 2E).

### 3.3. Immune-Related Gene Expression

**Figure 3:**
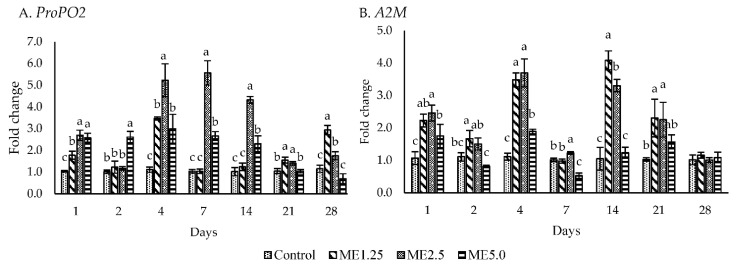
ProPO activating system. The effect of different concentrations of moringa extract in diet (control, ME1.25, ME2.5, and ME5.0) on the expression of *ProPO2* and *A2M* in the hemocytes of whiteleg shrimp. The data are expressed as mean ± standard deviation (*n* = 3). Bars with different letters at the same time point are significantly different (*p <* 0.05).

Figure 3A,B shows the gene expression of the ProPO activating system. The ME5.0 group exhibited an upregulated (*p* < 0.05) *ProPO2* expression from day 1 to day 14 and a downregulated *ProPO2* expression on day 28. ME2.5 demonstrated an upregulated *ProPO2* expression (*p* < 0.05) at all time points except for day 2. ME2.5 upregulated the *ProPO2* more than five times on days 4 and 7. A significant upregulation of *A2M* (*p* < 0.05) was observed on days 1, 4, 7, 14, and 21 in ME2.5 and on days 1 and 4 in ME5.0.

**Figure 4:**
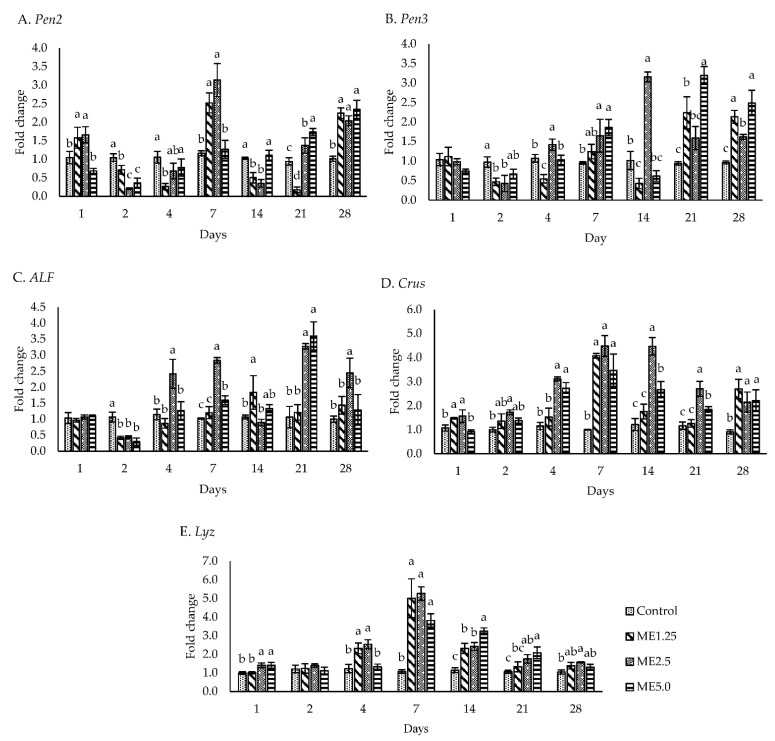
Antimicrobial peptides. The effect of different concentrations of moringa extract in diet (control, ME1.25, ME2.5, and ME5.0) on the expression of *Pen2*, *Pen3*, *ALF*, *Crus*, and *Lyz* in the hemocytes of whiteleg shrimp. The data are expressed as the mean ± standard deviation (*n* = 3). Bars with different letters at the same time point are significantly different (*p* < 0.05).

Five antimicrobial peptides were evaluated, including *Pen2*, *Pen3*, *ALF*, *Crus*, and *Lyz*. *Pen2* (Figure 4A) was significantly upregulated (*p* < 0.05) in the ME2.5 group on days 1, 7, 21, and 28, while *Pen3* (Figure 4B) was significantly upregulated on days 4, 7, 14 and 28. The ME5.0 group demonstrated *Pen3* expression on days 7, 21, and 28. The ME2.5 group exhibited an upregulated *ALF* expression (*p* < 0.05) on more days (days 4, 7, 21, and 28) compared with the ME5.0 (days 7 and 21) and ME1.25 (day 14) groups (Figure 4C). A significantly higher expression (*P* < 0.05) of *Crus* was observed in the ME2.5 group than in the control group on days 1 to 28, while *Crus* in ME5.0 was not upregulated on days 1 and 2 (Figure 4D). The ME1.25 group demonstrated an upregulated *Crus* expression (*p* *<* 0.05) on days 1, 7, and 28. The highest expression of *Lyz* in all the treatment groups was observed on day 7. Subsequently, *Lyz* expression gradually decreased until day 28 in all the treatment groups (Figure 4E). The ME2.5 group showed a significantly upregulated *Lyz* expression (*p* < 0.05) on days 1, 4, 7, 14, 21, and 28, whereas the ME5.0 group exhibited a significantly upregulated *Lyz* expression (*p* < 0.05) on days 1, 7, 14, and 21. *Lyz* was upregulated (*p* < 0.05) in the ME1.25 group on days 4, 7, and 14. Taken together, these findings (Figure 4) indicated that a high expression of these anti-microbial peptide genes was found on day 7, especially in the ME2.5 group.

**Figure 5:**
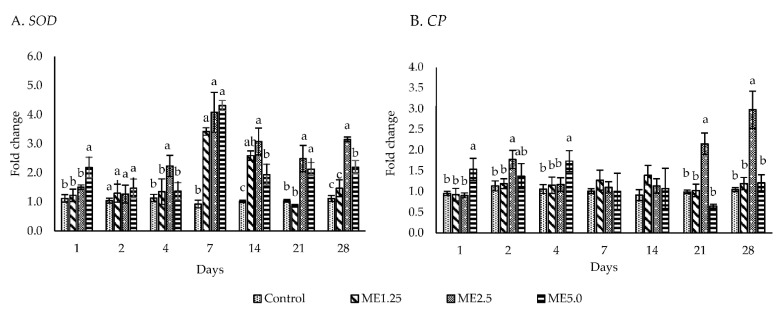
The effect of different concentrations of moringa extract in diet (control, ME1.25, ME2.5, and ME5.0) on the expression of *SOD* and *CP* in the hemocytes of whiteleg shrimp. The data are expressed as the mean ± standard deviation (*n* = 3). Bars with different letters at the same time point are significantly different (*p* < 0.05).

The expression of the antioxidant gene *SOD* was upregulated (*p* < 0.05) more than one time in the ME2.5 group from day 4 to 28 (Figure 5A). The ME1.25 group showed a significantly upregulated *SOD* expression (*p* *<* 0.05) only on days 7 and 14, whereas the ME5.0 group demonstrated a significantly upregulated *SOD* expression (*p* < 0.05) on days 1, 7, 14, 21, and 28. *CP* was not upregulated in the ME1.25 group (Figure 5B). However, the ME2.5 group revealed a significantly upregulated *CP* expression (*p* < 0.05) on days 2, 21, and 28, whereas the ME5.0 group showed a significantly upregulated *CP* expression (*p* < 0.05) only on day 4.

### 3.4. Growth Performance

During the 60-day rearing period, the growth performance of shrimp fed diets containing the moringa extract was higher than that of the control shrimp. The ME2.5 group showed a high weight gain, SGR, and a lower FCR (*p* < 0.05) than the control diet. However, it was not significantly different from that of the ME1.25 and ME5.0 groups. No significant difference in the survival rate was noted among the treatment groups (Table 3).

### 3.5. Challenge Test

**Figure 6:**
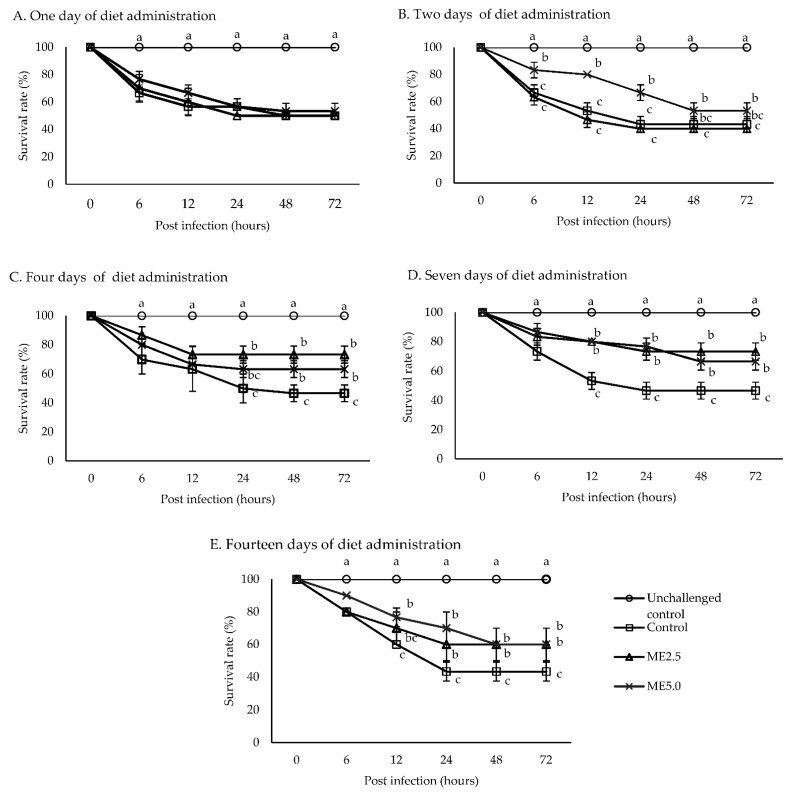
Survival rate during 72 h of whiteleg shrimp fed the dietary moringa extract (control, ME2.5, ME5.0) for 1, 2, 4, 7, and 14 days and then challenged with *V. alginolyticus*. The data are expressed as the mean ± standard deviation (*n* = 3). Bars with different letters at the same time point are significantly different (*p* < 0.05).

**Figure 7:**
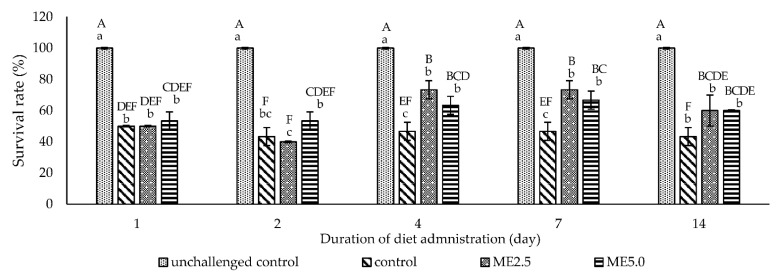
Cumulative survival rate at 72 h after challenge for all the different durations of diet administration (1, 2, 4, 7, and 14 days). The data are expressed as the mean ± standard deviation (*n* = 3). Bars with different lowercase letters (a–c) are significantly different (*p* < 0.05) at the same time point. Bars with different uppercase letters (A–F) are significantly different (*p* < 0.05) for all time points.

The unchallenged control group had a survival rate of 100%, while the survival rate of the other treatment groups dramatically decreased during 6 to 48 h after the injection of *V. alginolyticus*. The survival rate of the challenged controls was ranged from 43.3 to 50%. One day of diet administration had no effect on the survival rate of all the treatment groups (Figure 6A). After 2 days of diet administration, the survival rate of the ME5.0 group was significantly higher (*p* < 0.05) than that of the control group (Figure 6B). After 4 days of diet administration, the survival rate of the ME2.5 and ME5.0 groups was higher (*p* < 0.05) than that of the control group (Figure 6C). After 7 and 14 days of diet administration, the survival rates of the ME5.0 and ME2.5 groups were significantly higher (*p* < 0.05) than that of the control group (Figure 6D,E). Overall, 72 h after the bacterial injection, the survival rate was significantly high (*p* < 0.05) compared to the control in the ME2.5 group fed the treatment diet for 4 and 7 days (73.3%), followed by the ME5.0 group fed the treatment diet for 7 days (66.7%) (Figure 7).

## 4. Discussion

### 4.1. In Vitro Study of Viability and Immune Response

The water extract of some herbs has been proven to increase the immune response of whiteleg shrimp [28,29,39,40]. Moringa herb has antitumor, antioxidant, anticancer, antistress, immunostimulatory effects in gilthead seabream (*Sparus aurata*) and nile tilapia [41,42,43,44,45], because of its phytochemicals, such as phenolic acid, flavonoids, carotenoids, alkaloids, tannin, lectin, and terpenoids [46].

The results of this study revealed that the viability of the hemocytes treated with up to 250 ppm of the moringa extract was the same as that of the control group; however, the viability tended to decrease in hemocytes treated with a dosage of more than 250 ppm. Thus, the moringa extract is safe for hemocytes at a low dosage, of up to 250 ppm. Assessing toxicity is essential, as it may directly affect the organism—for example, through membrane disruption [47] and hematological deterioration [48]. Some active compounds of the plant, such as saponins and tannins, induced cytotoxic effects when present in a high dosage but triggered an immune function when present in a low dosage, as found in Zebrafish (*Danio rerio*) [49], *Lateolabrax maculatus* [50], and *Lateolabrax japonicus* [51]. A moringa water extract showed a cytotoxic effect on cells of gilthead seabream at 250 to 1000 ppm [42].

The in vitro assay revealed that the moringa extract increased the PO activity, PR, and superoxide anion production at the dosage of 100 to 250 ppm. The in vitro assay was performed to analyze the effect of immunostimulants on the shrimp hemocytes. The in vitro study is a cost-effective alternative to select new immunostimulants candidates for aquaculture, as they may yield more repetitive results under a highly controlled experiment environment. These immune parameters did not directly increase when the moringa extract concentration was increased above 500 ppm. The in vitro assay results propose that the moringa extract may be used as a feed additive for whiteleg shrimp.

### 4.2. In Vivo Study of Immune Responses

A high count of circulating hemocytes is essential for the shrimp’s immune system [52]. All dietary moringa has shown effectiveness in increasing the THC of the whiteleg shrimp associated with the treated days and concentration. Since THC is linked with the resistance of whiteleg shrimp against vibriosis infection [53], the moringa extract is therefore thought to enhance the resistance of the shrimp against disease caused by the bacteria.

PO activity is commonly used as an indicator to evaluate the potential effect of herb extracts on shrimp immunity. The PO enzyme is involved in the hemolymph melanization. The in vivo assay revealed that the moringa extract, particularly in the ME2.5 group, improved the PO activity from day 2 to day 28. The expression of *ProPO2* in the ME2.5 group reached its maximum on days 4 and 7, whereas *A2M* expression was downregulated at the same time points. Ponprateep et al. [54] found that the *A2M* activity was decreased, whereas the PO activity was increased to balance the melanin synthesis for maintaining the host’s homeostasis. This study showed that moringa and its components trigger the activation of ProPO cascades in the shrimp, especially in the ME2.5 group. Akbary et al. [14] found that dietary moringa induced PO activity in whiteleg shrimp at 0.5 to 1.5 g per kg of diet.

Phagocytosis is stimulated by the components of the putative ProPO-activating system. PO is a sticky protein that attaches to various surfaces of pathogens, such as fungi [55] that enhance phagocytosis [56,57]. During phagocytosis, many anti-microbial substances that are essential for eliminating bacteria, such as superoxide anions, are produced. Phagocytosis is correlated with reactive oxygen species, such as superoxide anions, that are highly microbiocidal [58].

The results of the in vivo assay indicated that the moringa extract resulted in a high PR and PI on day 7 in the ME2.5 group, which was accompanied by the high generation of superoxide anions. An excessive superoxide anions production may cause oxidative stress, that can damage cells [59]. To prevent this damage, the cells generate SOD. Wu et al. [60] reported that SOD activation is based on superoxide anion production.

On day 7, the ME2.5 and ME5.0 groups showed a high expression of *SOD*; however, this expression decreased in the following days and was stable on days 21 and 28. This might be associated with the superoxide anion production, that declined during the same period. Previous studies showed that SOD was increased in shrimp [14] and nile tilapia [61] fed a dietary moringa extract at 1 g and 0.4 to 0.5 g per kg of diet, respectively

Antibacterial peptides were upregulated predominantly in the ME2.5 group on day 7. Anti-microbial peptides exhibit a broad spectrum of activity against gram-positive and gram-negative bacteria, yeast, fungi, parasites, enveloped viruses, and even tumor cells [62]. Penaedins are unique anti-microbial peptides present in shrimp and consist of three classes—*Pen2*, *Pen3*, and *Pen4*—that are distinguished by the N-terminal proline-rich domain and the C-terminal cysteine-rich domain [63]. Penaedins are active against gram-positive bacteria and fungi [64,65]. Wang et al. (2010) reported that *Pen3* does not participate in antivibrio immune responses; however, *Pen2* was upregulated in cells after a negative bacterial injection (*V. harveyi*) [37]. *ALF* is involved in inhibiting the infection of filamentous fungi and gram-positive and gram-negative bacteria and is highly efficient against vibrio species [66]. *Crus* plays a vital role in vibrio infection in shrimp and was upregulated after the administration of vibrio in shrimp [37,67]. *Crus* participates in the apoptosis and phagocytosis of shrimp hemocytes [68], and its absence in shrimp infected by vibrio may significantly increase their mortality [69]. In the present study, *Lyz* was immediately expressed from day 1 to day 28. Another study demonstrated that nile tilapia fed a diet containing 1.5% of moringa for 60 days showed a significant increase in lysozyme activity [15]. *Lyz* can effectively inhibit gram-positive bacteria because of its ability to cleave β-1,4 glycosidic bonds between N-acetylmuramic acid and N-acetylglucosamine in the cell wall layer of bacteria [70,71]. The expression of *Pen2, Pen3, ALF, Crustin,* and *Lysozyme* during the experiment indicated that the moringa extract, especially at ME2.5, can potentially control the infection of fungi, gram-positive, and gram-negative bacteria.

*CP* is involved in the coagulation of the hemolymph of crustaceans, which prevents the loss of body fluids and the entry of opportunistic pathogens [72]. *CP* is a critical molecule that prevents bacterial and viral infections [73]. In the present study, *CP* was upregulated, but only on some days during treatment and in different concentrations of the moringa extract. It is indicated that the moringa extract enhances shrimp’s defense mechanism against the pathogen.

### 4.3. Growth Performance

The existence of antinutrients in moringa could severely interfere with the absorption of nutrients in the diet, which finally depressed the growth [74]. Dietary moringa at ME2.5 exhibited a significant effect on the growth performance of whiteleg shrimp, but at a high concentration (ME5.0) revealed no significant effect on growth. Studies showed that a dietary supplementation with moringa at a particular range concentration improved the growth performance of some aquaculture species [46]. A high concentration of moringa extract in diet might inhibit digestive enzymes and complex dietary proteins because of the presence of tannins, saponins, and other secondary metabolites, which do not provide nutrition when present in unacceptable amounts [75,76]. However, when present in appropriate amounts, the incorporation of herb extracts in aquatic animals’ diet stimulated the secretion of digestive enzymes, protease, amylase, and lipase, and acted as an appetizer, thus improving the growth and diet utilization [77]. The present study showed that the sharing of the antinutritional factor at 2.5 g of moringa water extract per kg of diet was still acceptable for a better growth and diet utilization in whiteleg shrimp. However, another study reported that whiteleg shrimp fed a diet containing a moringa leaves’ extract at 1 g per kg showed a positive effect on growth performance [14]. In addition, giant freshwater prawn fed a diet containing a moringa extract at 2.5 to 5.0 g demonstrated a better growth performance than the control [13].

### 4.4. Challenge Test

The challenge test was carried out to investigate the increasingly observed immune parameter in vitro and in vivo associated with the resistance of the whiteleg shrimp against *V. alginolyticus*. This study shows that the highest immune responses did not occur on a particular day but varied during the experiment. Therefore, the vibrio injection was performed after the shrimp fed dietary moringa for 1, 2, 4, 7, and 14 days. The result showed that the administration of a diet containing the moringa extract for 2 until 14 days increased the survival rate of the shrimp after challenging *V. alginolyticus*. A high survival rate was caused by ME2.5 administration for 4 and 7 days, followed by ME5.0 administration for 7 days compared to the control group. An increased survival for day 7 of diet administration in the ME2.5 and ME5.0 groups was associated with immune parameters, including *ProPO2*, anti-microbial peptide gene expression, *SOD*, PR as well as superoxide anion production, which were also high on day 7. In accordance with the findings of previous studies, herb administration resulted in a high immune response after approximately 1 week of feeding [28,29,40,78].

## 5. Conclusions

In conclusion, this study confirmed that the positive immune response in vitro and in vivo and the immune-related gene expressions were differently enhanced according to the moringa concentration in the diet and feeding duration. The dietary moringa extract enhanced the THC, PO activity, PR, PI, and superoxide anion production. In addition, dietary moringa upregulated the gene expressions of proPO activating systems, antibacterial peptides, superoxide dismutase, and clotting protein. Moringa supplementation at 2.5 g per kg resulted in the most favorable improvement of nonspecific immune responses and growth performances of whiteleg shrimp. In addition, the administration of a diet containing moringa at 2.5 g per kg for 4 and 7 days was effective against the *V. alginolyticus* infection.

## Figures and Tables

**Table 1:** Ingredients and proximate diet compositions.

Ingredients (g per kg Diet)	Control	ME1.25	ME2.5	ME5.0
Fish meal	500	500	500	500
Shrimp meal	60	60	60	60
Yeast	50	50	50	50
Moringa extract	0	1.25	2.5	5
a-starch	150	150	150	150
fish oil	15	15	15	15
Lecithin	5	5	5	5
Cholesterol	5	5	5	5
Choline chloride	5	5	5	5
Vitamin D3	1	1	1	1
Vitamin E	1	1	1	1
Vitamin A	1	1	1	1
Vitamin premix ^1^	40	40	40	40
Mineral premix ^2^	40	40	40	40
a-cellulose	127	125.75	124.5	122
Proximate analysis (% in dry weight)				
Crude protein	50.11	50.63	50.65	50.84
Crude lipid	8.79	8.90	8.78	8.74
Ash	11.45	11.28	11.57	11.74
Moisture	7.8	7.9	7.5	7.2

^1^ The mineral premix includes 2.1% calcium carbonate, 73.5% calcium phosphate dibasic, 0.227% citric acid, 0.046% cupric acid, 0.558% ferric acid (16–17% Fe), 2.5% magnesium oxide, 0.825% magnesium citrate, 6.8% potassium iodide sulphate, 3.06% sodium chloride, 2.14% sodium phosphate, 0.133% zinc citrate, 0.001% potassium iodine, and 8.1% potassium phosphate dibasic. ^2^ The vitamin premix includes 0.5% thiamin HCl, 0.8% riboflavin, 2.6% niacinamide, 0.1% D-biotin, 1.5% Ca-pantothenate, 0.3% pyridoxin HCl, 0.5% folic acid, 18.1% inositol, 3% para-aminobenzoic acid, 0.1% cyanocobalamin, 0.1% BHT, 60.3% a-cellulose, and 12.1% ascorbic acid.

**Table 2:** Primers of immune-related genes.

Gene	Primer Name	Sequence (5′-3′)	References
Prophenoloxidase2	proPO2-F proPO2-R	ACCACTGGCACTGGCACCTCGTCTA TCGCCAGTTCTCGAGCTTCTGCAC	[36]
α2macroglobulin	A2M-F A2M-R	GCACGTAATCAAGATCCG CCCATCTCATTAGCACAAAC	[36]
Penaiedin2	Pen2-F Pen2-R	TCGTGGTCTGCCTGGTCTT CAGGTCTGAACGGTGGTCTTC	[37]
Penaiedin3	Pen3-F Pen3-R	CACCCTTCGTGAGACCTTTG AATATCCCTTTCCCACGTGAC	[37]
Anti-LPS factor	*ALF*-F *ALF*-R	CTGTGGAGGAACGAGGAGAC CCACCGCTTAGCATCTTGTT	[37]
Crustin	*Crus*-F Crus-R	GAGGGTCAAGCCTACTGCTG ACTTATCGAGGCCAGCACAC	[37]
Lysozyme	*Lyz*-F *Lyz*-R	GAAGCGACTACGGCAAGAAC AACCGTGAGACCAGCACTCT	[37]
Superoxide dismutase	*SOD*-F SOD-R	ATCCACCACACAAAGCATCA AGCTCTCGTCAATGGCTTGT	[38]
Clotting protein	*CP*-F *CP*-R	TCTTTGCGCAGTTGGTGATC TGAGGTGACCGAGTGCAAAA	[37]
EF1α	*EF1α* F *EF1α*R	ATGGTTGTCAACTTTGCCCC TTGACCTCCTTGATCACACC	[36]

**Table 3:** The growth performance of whiteleg shrimp fed dietary moringa extracts with different concentrations (control, ME1.25, ME2.5, and ME5.0) for 60 days.

Diet	Initial Weight (g)	Final Weight (g)	Weight Gain (%)	SGR (% Day^−1^)	FCR	Survival Rate
Control	0.59 ± 0.05	5.57 ± 0.35 ^b^	855 ± 136 ^b^	3.74 ± 0.24 ^b^	1.39 ± 0.01 ^a^	91.67 ± 7.22
ME1.25	0.59 ± 0.05	6.50 ± 0.57 ^ab^	1018 ± 183 ^ab^	4.01 ± 0.28 ^ab^	1.35 ± 0.03 ^ab^	91.67 ± 7.22
ME2.5	0.54 ± 0.01	6.96 ± 0.47 ^a^	1200 ± 81 ^a^	4.26 ± 0.11 ^a^	1.29 ± 0.01 ^c^	91.67 ± 7.22
ME5.0	0.56 ± 0.02	6.39 ± 0.10 ^ab^	1039 ± 40 ^ab^	4.04 ± 0.08 ^ab^	1.34 ± 0.02 ^bc^	95.83 ± 7.22

The data are presented as the mean ± SD. Means in the same column with different superscripts are significantly different (*p* < 0.05). SGR, specific growth rate; FCR, feed conversion ratio.

## Data Availability

The authors confirm that the data supporting the findings of this study are available within the article.
